# Finding, treating and retaining persons with HIV in a high HIV prevalence and high treatment coverage country: Results from the Botswana Combination Prevention Project

**DOI:** 10.1371/journal.pone.0250211

**Published:** 2021-04-21

**Authors:** Pamela Bachanas, Mary Grace Alwano, Refeletswe Lebelonyane, Lisa Block, Stephanie Behel, Elliot Raizes, Gene Ussery, Huisheng Wang, Faith Ussery, Molly Pretorius Holme, Connie Sexton, Sherri Pals, Arielle Lasry, Lisetta Del Castillo, Shannon Hader, Shahin Lockman, Naomi Bock, Janet Moore

**Affiliations:** 1 Division of Global HIV/AIDS, U.S. Centers for Disease Control and Prevention, Atlanta, Georgia, United States of America; 2 Division of Global HIV/AIDS, U.S. Centers for Disease Control and Prevention, Gaborone, Botswana; 3 Botswana Ministry of Health and Wellness, Gaborone, Botswana; 4 Northrup Grumman, Atlanta, Georgia, United States of America; 5 Harvard T.H. Chan School of Public Health, Boston, Massachusetts, United States of America; 6 UNAIDS, Geneva, Switzerland; 7 Botswana Harvard AIDS Institute Partnership, Gaborone, Botswana; 8 Brigham and Women’s Hospital, Boston, Massachusetts, United States of America; IRCCS Neuromed, CUAMM, ITALY

## Abstract

**Introduction:**

The scale-up of Universal Test and Treat has resulted in reductions in HIV morbidity, mortality and incidence. However, healthcare system and personal challenges have impacted the levels of treatment coverage achieved. We implemented interventions to improve linkage to care, retention, viral load (VL) coverage and service delivery, and describe the HIV care cascade over the course of the Botswana Combination Prevention Project (BCPP) study.

**Methods:**

BCPP was designed to evaluate the impact of prevention interventions on HIV incidence in 30 communities in Botswana. We followed a longitudinal cohort of newly identified and known HIV-positive persons not on antiretroviral therapy (ART) identified through community-based testing activities through BCPP and referred with appointments to local HIV clinics in 15 intervention communities. Those who did not keep the first or follow-up appointments were tracked and traced through phone and home contacts. Improvements to service delivery models in the intervention clinics were also implemented.

**Results:**

A total of 3,657 newly identified or HIV-positive persons not on ART were identified and referred to their local HIV clinic; 90% (3,282/3,657) linked to care and of those, 93% (3,066/3,282) initiated treatment. Near the end of the study, 221 persons remained >90 days late for appointments or missing. Tracing efforts identified 54/3,066 (2%) persons who initiated treatment but died, and 106/3,066 (3%) persons were located and returned to treatment. At study end, 61/3,066 (2%) persons remained missing and were never reached. Overall, 2,951 (98%) persons living with HIV (PLHIV) who initiated treatment were still alive, retained in care and still receiving ART out of the 3,001 persons alive at the end of the study. Of those on ART, 2,854 (97%) had current VL results and 2,784 (98%) of those were virally suppressed at study end.

**Conclusions:**

This study achieved high rates of linkage, treatment initiation, retention and VL coverage and suppression in a cohort of newly identified and known PLHIV not on ART. Tracking and tracing interventions effectively identified those persons who needed more resource intensive follow-up. The interventions implemented to improve service delivery and data quality may have also contributed to high linkage and retention rates.

**Clinical trial number**: NCT01965470.

## Introduction

The widespread scale-up of antiretroviral therapy (ART) for persons living with HIV (PLHIV) in low- and middle-income countries has contributed to reductions in morbidity, mortality and new HIV infections [1]. However, failure to engage all PLHIV in care and retain them on treatment due to personal (e.g., disclosure or privacy concerns) or healthcare system (e.g., frequent appointments, long wait times) challenges have impacted the levels of treatment coverage achieved [2–7]. Recent studies on Universal Test and Treat (UTT) have highlighted the importance of linking and retaining clients on treatment and providing client-centered care to reach the high levels of treatment coverage and viral suppression needed for epidemic control [8–12].

The HIV care cascade has been used widely to assess progress in reaching the UNAIDS 90-90-90 targets [13], as well as to identify gaps in HIV programs [14]. The cascade describes the benchmark stages along the pathway to viral suppression including HIV testing, knowledge of HIV status, linkage to care, ART initiation, retention in care, and viral load (VL) suppression [15]. Tracking individuals through the cascade longitudinally provides an accurate assessment of loss at each step, informs implementation of targeted interventions to address identified gaps, and enables assessment of uptake of intervention strategies and progress toward 90-90-90 goals [7, 16–21].

With the scale-up of testing and treatment in recent years, the need to strengthen linkage and retention support to PLHIV and address weaknesses in healthcare delivery systems has become more evident [22]. Strategies that track and trace persons who fail to link to care or are late for clinic visits or drug pick-ups have shown success in returning some patients to care and documenting outcomes on others [5, 23, 24]. Reducing the number of clinic visits, rapid or same-day ART initiation, and supportive interventions including peer counseling have also been shown to reduce losses along the cascade [25–27]. However, these interventions have not yet been implemented at scale in most settings in Sub-Saharan Africa.

Botswana was one of the first countries in Africa to offer free ART to its citizens beginning in 2002 and to implement UTT in 2016 and has made significant progress scaling-up these key interventions [28]. Yet, Botswana has the second highest HIV prevalence in the world (estimated at 22% of adults) [29]. The Botswana Combination Prevention Project (BCPP) implemented a combination of community-based HIV testing initiatives and expanded ART provision in 15 intervention communities. Interventions to improve linkage to care, retention, VL coverage, service delivery and monitoring of the HIV care cascade were also implemented. The aim of this paper is to describe the HIV care cascade in a longitudinal cohort over the course of the study, documenting individual level outcomes along each step. Interventions implemented to achieve and sustain these outcomes are also described.

## Methods

**Fig 1:**
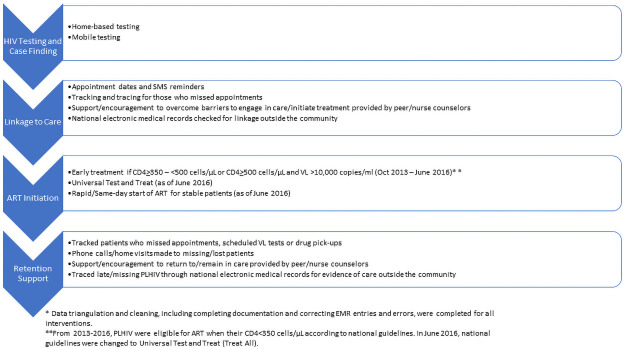
BCPP interventions across the clinical cascade.

BCPP was a cluster-randomized trial that evaluated the impact of prevention interventions on population-level HIV incidence in 30 communities. A full description of the study design is available elsewhere [28]. The sub-analyses reported here are from a longitudinal cohort of PLHIV not on ART identified through community testing activities in the 15 intervention communities. The interventions were delivered in government clinics by the Ministry of Health and Wellness (MoHW) staff and local implementing partners and took place from October 2013 through March 2018 (Fig 1). The intervention clinics were in rural or peri-urban communities with an average size of 6,000. All persons 16 years or older who were newly identified as HIV-positive or were HIV-positive but not on ART identified through community testing activities in the intervention communities were eligible for study interventions, if they were community residents (spent at least three nights/month in the community) and Botswana citizens or spouses of citizens. Most Botswana citizens have a unique individual identification number (Omang) issued by the government which is used by the healthcare system.

### HIV testing

With the goal of 100% of PLHIV in the community knowing their status, home-based and mobile HIV testing and case-finding of known PLHIV were conducted with persons >16 years over the course of the study. Individuals who agreed to participate completed a brief demographic questionnaire (standard questionnaire administered prior to testing by the community partner); persons who knew their HIV-positive status and had documentation were identified and those who did not have documentation of being HIV-positive or did not know their status and accepted HIV testing services provided verbal consent for testing. Verbal consent for completing the demographic questionnaire and HIV testing is the standard in Botswana, and the age of consent for testing is 16. Therefore, written consent was not required and verbal consent was documented by the interviewer/counselor on the electronic version of the form. Point-of-care fingerstick testing was conducted according to the Botswana national testing algorithm. Samples from each community were retested in the reference laboratory to provide quality control for the field-based HIV rapid testing [30]. The first community used the enzyme-linked immunosorbent assay (ELISA) using the Vironostika Uniform II Plus O (bioMerieux, Marcy l’Etoile, France), and subsequent communities used the Bio-Rad Genetic Systems^™^ HIV-1/HIV-2 *PLUS O* EIA (Bio-Rad Laboratories, Redmond, WA, USA) in accordance with the Botswana national HIV testing algorithm. In addition, starting in June 2016, all persons referred with a positive HIV test underwent verification HIV rapid testing at the clinic.

### Linkage to HIV care and ART

All persons newly identified or known HIV-positive but not on ART identified through community testing activities were given referral forms with specific appointment dates at their local MoHW clinics (appointments are not a usual practice in Botswana). A Linkage Coordinator at each local MoHW HIV clinic sent SMS appointment reminders the day before their appointment. Using participants’ names and Omang numbers, Linkage Coordinators tracked participants through the MoHW’s electronic medical records systems (EMRs) and Clinic Registration Log to determine if they kept their scheduled appointment. Persons who were seen for an HIV service at their local clinic were considered linked. Participants who did not keep their appointment were traced by a community-based peer counselor through alternating phone and home contacts and were provided support with issues such as disclosure and navigating the clinic.

For participants who did not link at their local clinic, searches were conducted using the Omang number in the EMRs and laboratory database to determine if they were receiving HIV services at a clinic outside their community of residence. When participants were found at a clinic outside their local community, a nurse from the clinic in the participant’s community of residence called and verified that the participant was currently enrolled in care and on ART. All persons who were verified on ART at another clinic were counted as linked for analysis purposes.

### ART initiation

From 2013–2016, PLHIV were eligible for ART when their CD4<350 cells/μL according to national guidelines. However, study participants in the intervention communities were eligible for early treatment through BCPP if they had CD4>350 –<500 cells/μL or CD4>500 cells/μL and VL >10,000 copies/ml. From mid-2016, all PLHIV were eligible for ART regardless of CD4 count, as the government of Botswana adopted UTT.

Rapid (within 7 days) and same-day ART initiation [31] was also started in 2016 at the intervention sites with patients who were clinically stable (WHO stage I or II and no opportunistic diseases) starting treatment the day of diagnosis or their first clinic visit. The implementation of Rapid/Same-day start included reducing the number of pre-ART visits, not waiting for the return of baseline labs before initiating treatment and calling back patients with abnormal lab values. Stable patients (virally suppressed, no opportunistic infections, and keeping appointments) who were on ART longer than 12 months were also eligible for multi-month dispensing and received 2–3 months of medications per pick-up, as opposed to the standard monthly medication pick-up.

### Viral load testing

Viral load testing was conducted at three months post-ART-initiation and every six months thereafter. Viral suppression was defined as <400 copies/ml. Interventions such as contacting persons with late or missing VL tests and rescheduling appointments, ensuring VL results were entered in patients’ charts and EMRs, and reducing sample transport time to the lab and turn-around-time of results were implemented to improve VL coverage.

### Retention in care/on treatment

Linkage coordinators and clinic nurses reviewed weekly and monthly clinic reports generated by the EMRs of PLHIV who missed clinic appointments, drug pick-ups or VL tests or who were lost to follow up (LTFU; missing from the clinic >90 days). Tracking and tracing through phone calls by clinic staff and counselors and home visits by counselors for those unable to be reached by phone were conducted to reschedule appointments and support re-enrollment/re-initiation on treatment when indicated. Tracing missing PLHIV through EMRs and other data systems was also conducted, which included finding many persons still active on ART through data cleaning efforts, correcting unique identifiers, and locating patients on treatment at other outside clinics. Using the Omang and line lists, patients who were still active in care but had not been entered into EMRs or lab systems correctly were identified by triangulating patient charts, EMR and labs. Near the end of the study, there was an intensified effort to track LTFU patients which included all of the above, contacting family members and documenting deaths and LTFU. This required clinic staff and Linkage Coordinators dedicating focused time and effort on tracing activities.

### Data collection and statistics

All questionnaires and results of HIV tests were collected on encrypted handheld tablets, which were synchronized daily with the research database. Participants were tracked through the HIV care cascade and de-duplicated using the Omang. Whether participants kept appointments or received HIV services, as well as clinical indicators such as treatment initiation and VL, were abstracted from the clinics’ EMRs. These data were merged with the questionnaire data collected in the community for a continuous record on each participant from identification through retention on ART.

SAS 9.4 (SAS Institute Inc., Cary, NC) was used for all statistical analyses. SAS PROC GLIMMIX was used to test for association between sociodemographic, new or known HIV-positive status and linkage to care. Models included a random effect for community to adjust standard errors for within-community correlation, and all candidate variables were included in univariate and multiple regression models. P-values of less than 0.05 were considered statistically significant, and unadjusted and adjusted odds ratios (ORs) and 95% confidence limits were estimated.

### Ethics

The study was approved by the U.S. Centers for Disease Control and Prevention Institutional Review Board (Protocol #6475) and the Botswana Health Research and Development Committee (Institutional Review Board of the Botswana MoHW HPDME 13/18/1).

## Results

### Identification of PLHIV

From October 2013 to December 2017, 61,655 people with unique identifiers were assessed for HIV status (were tested or provided documentation of HIV-positive status or of HIV-negative test within past 3 months) in the 15 intervention communities. Twenty-two percent (13,328/61,655) of the persons assessed were HIV-positive; 26% (8,981/33,961) of women and 16% (4,347/27,694) of men. Of the 13,328 persons identified with HIV, 85% (11,288) already knew their status and had documentation thereof, and 15% (2,040) were newly identified through testing in BCPP. Persons newly testing HIV-positive (n = 2,040) and persons knowing their HIV-positive status but who were not on ART (n = 1,617) resulted in 3,657 or 27% of all 13,328 PLHIV identified through BCPP. These 3,657 PLHIV were referred to their local HIV clinic for ART initiation.

### Demographics of PLHIV referred to treatment

Of the 3,657 referred for treatment, 61% were female. Fifteen percent of referred persons were between 16 and 24 years of age, 34% between 25 and 34, and 51% between 35 and 64 (Table 1). Fifty-six percent (2,040/3,657) were newly identified as HIV-positive during the study and 44% (1,617/3,657) knew their HIV-positive status prior to the study but were not on ART. Sixty-six percent of persons referred reported secondary or higher education, and 45% were employed. Most people (73%) were single or never married, 24% were married or co-habiting, and 3% divorced or separated.

**Table 1:** Demographic characteristics of PLHIV referred to treatment.

	Persons with HIV Not on ART*		
	Identified through BCPP**		
	Total	Female	Male
	3,657	2,217 (61%)	1,440 (39%)
**Age (years)**			
16–24	542 (15%)	422 (19%)	120 (8%)
25–34	1,253 (34%)	777 (35%)	476 (33%)
35–64	1,862 (51%)	1,018 (46%)	844 (59%)
**New or Known HIV-Positive Status**			
Newly Identified HIV-Positive	2,040 (56%)	1,063 (48%)	977 (68%)
Known HIV-Positive Not on ART	1,617 (44%)	1,154 (52%)	463 (32%)
**Education Level**	n = 3,655	n = 2,217	n = 1,438
Primary or Lower	1,235 (34%)	680 (31%)	555 (39%)
Secondary or Higher	2,420 (66%)	1,537 (69%)	883 (61%)
**Employment Status**	n = 3,630	n = 2,195	n = 1,435
Employed (Full/Part Time)	1,639 (45%)	846 (39%)	793 (55%)
Unemployed	1,991 (55%)	1,349 (61%)	642 (45%)
**Relationship Status**	n = 3,656	n = 2,216	n = 1,440
Single/Never Married	2,655 (73%)	1,645 (74%)	1,010 (70%)
Cohabitating/Married	880 (24%)	479 (22%)	401 (28%)
Divorced/Separated/Widowed	121 (3%)	92 (4%)	29 (2%)

*ART = Antiretroviral therapy;

**BCPP = Botswana Combination Prevention Project.

### Linkage to care of persons referred to treatment

Of the 3,657 people referred to treatment, 3,282 (90%) linked to care (i.e., received an HIV-related service at an MoHW clinic). The 3,282 persons who linked to care were compared to the characteristics (i.e. sex, age, education, employment, new or known HIV-positive status and marital status) of the 375 (10%) persons referred to treatment who never linked (Table 2). All factors were significantly related to linkage in the univariate analyses; however, age was no longer a significant predictor in the multivariate analysis. A lower percentage of males (86%) than females (92%) linked to care (p < .0001). A lower percentage of persons with a secondary or higher education linked (88%) than did persons with only primary education (94%; p < .0001), and a lower percentage of persons who were employed (87%) compared to those who were not employed (92%; p = .0066). A lower percentage of PLHIV who were newly identified linked (88%) than did those who already knew their HIV-positive status (92%; p = .0061). In addition, married or cohabitating persons were less likely to link than single people (p = .0237). Consistent with these findings, the most frequently self-reported reasons for not keeping appointments by persons who were seen by counselors during tracing but never linked included (from most frequently reported to less) 1) being too busy to attend clinic, 2) being unable to miss school/work, 3) lack of disclosure to family/sex partner, 4) receiving care/treatment at clinic outside study community, 5) not ready to accept HIV status, and 6) afraid of seeing family or community members at clinic.

**Table 2:** Univariate and multivariate analyses of the association of sociodemographic variables with linkage to care.

Variable	Linked	Unadjusted OR	P	Adjusted OR	p
	N (%)	(95% CI)		(95% CI)	
**Sex**			<0.0001		<0.0001
Female	2045 (92)	Ref		Ref	
Male	1237 (86)	0.51 (0.41, 0.64)		0.54 (0.43, 0.68)	
**Age**			0.0282		0.1445
16–24	477 (88)	Ref		Ref	
25–34	1107 (88)	1.00 (0.73, 1.38)		1.20 (0.86, 1.68)	
35–64	1698 (91)	1.35 (0.99, 1.83)		1.42 (0.99, 2.04)	
**Education**			<0.0001		<0.0001
Primary or lower	1158 (94)	2.02 (1.55, 2.63)		1.97 (1.47, 2.64)	
Secondary or higher	2122 (88)	Ref		Ref	
**Employment status**			<0.0001		0.0066
Employed	1426 (87)	0.64 (0.51, 0.80		0.72 (0.57, 0.91)	
Not employed	1832 (92)	Ref		Ref	
**New or Known HIV-Positive Status**			<0.0001		0.0061
Newly Identified HIV-Positive	1793 (88)	Ref		Ref	
Known Positive, not on ART	1489 (92)	1.61 (1.28, 2.02)		1.39 (1.10, 1.77)	
**Marital Status**			0.0396		0.0237
Single/Never Married	2396 (90)	Ref		Ref	
Cohabitating/Married	770 (88)	2.02 (0.88, 4.64)		0.71 (0.55, 0.91)	
Divorced/Separated/Widowed	115 (95)	1.66 (0.72, 3.83)		1.13 (0.48, 2.67)	

Of the 3,282 (90%) PLHIV who linked to care, 55% (1,809) kept the appointment in the clinic in their community of residence, and 34% (1099) linked later at that clinic. Eleven percent (374) of referred persons linked to care at MoHW clinics outside their community of residence. For participants who missed their first clinic appointment, an average of two home visits or telephone contacts were made by the linkage counselor before participants were seen at their respective clinics.

### ART initiation and retention among persons linking to care

**Fig 2:**
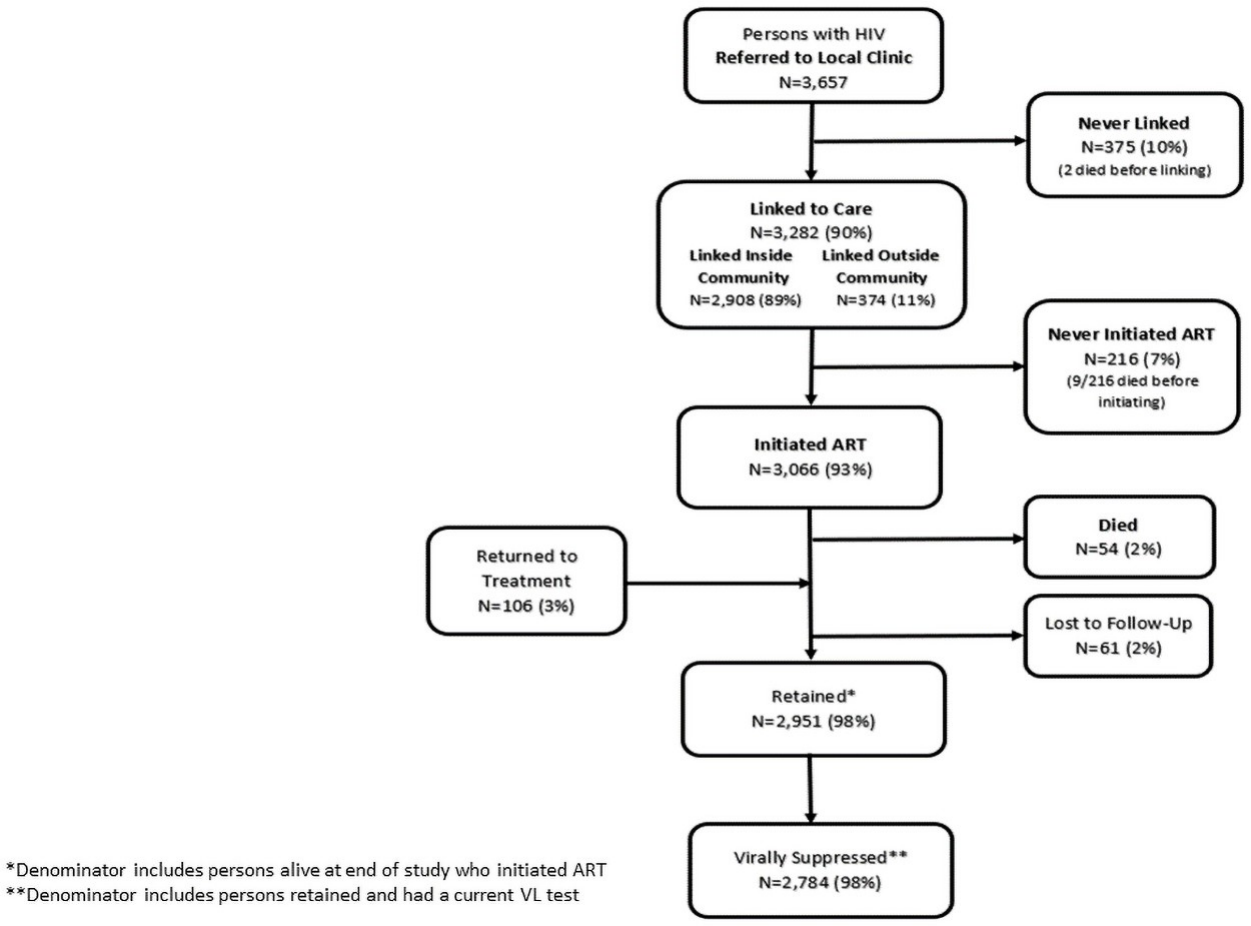
Linkage, ART initiation and retention outcomes among persons referred for care and treatment in intervention communities.

Ninety-three percent (3,066) of the 3,282 persons who linked to care initiated treatment, which represents 84% of those referred initiated treatment. Of the 216 persons who linked but did not initiate treatment, 9 (4%) died before initiating ART. During the course of the study, 221 persons were missing and/or >90 days late picking up their ART. Tracing efforts identified 54/3,066 (2%) persons who had initiated treatment but died, and 61/3,066 (2%) persons who were never reached/located and remained LTFU at study end. One hundred and six (3%) persons were located and returned to treatment (Fig 2). Overall, 2,951 (98%) PLHIV who initiated treatment were still alive, retained in care and receiving ART at the end of the study out of the 3,012 persons alive at the end of the study.

### Viral load testing and viral suppression

Among the 2,951 patients retained on ART, 2,854 (97%) had a current VL test at study end. During the study, 862 of the 2,951 people retained on ART were missing current VLs. Of those, 765 patients (88%) were successfully traced and returned to the clinic for updated VL testing, or their laboratory records were obtained with a current VL result. Of the 2,854 who had a current VL test at study end, 2,784 (98%) were virally suppressed. With an assumption that the 97 persons without a VL test were not virally suppressed, the VL suppression rate was 94% at study end.

### Clinical cascade results in the referral cohort

**Fig 3:**
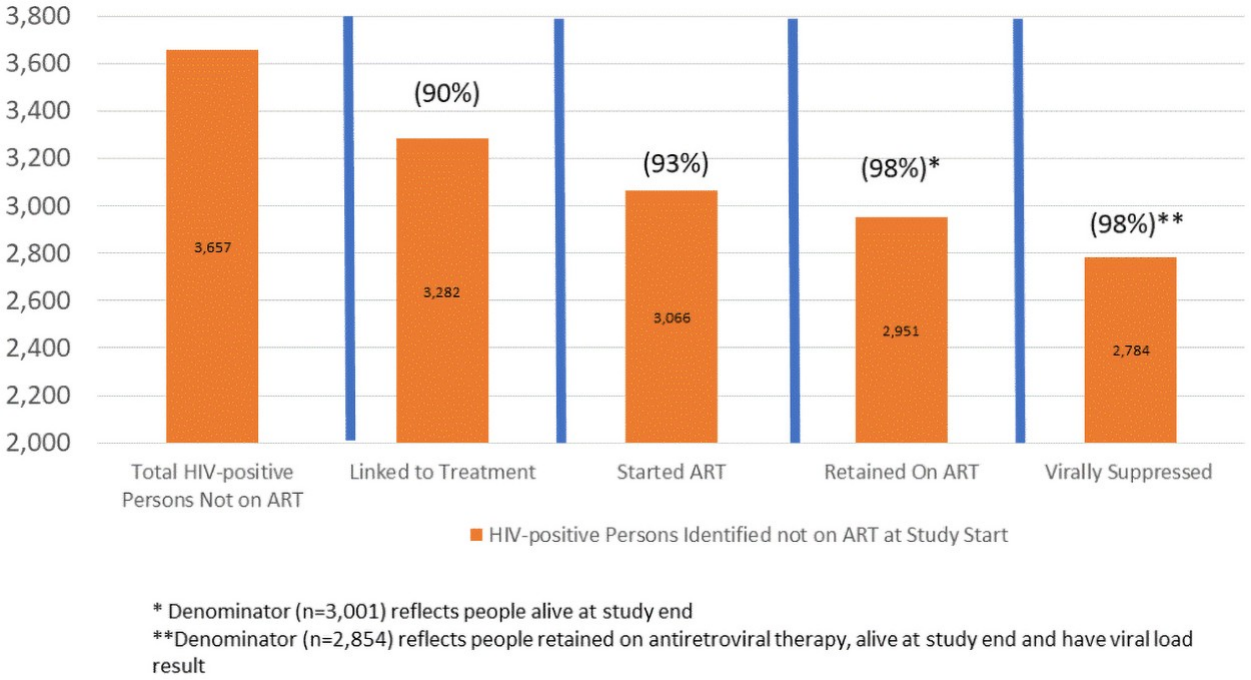
Longitudinal HIV care cascade among PLHIV not on ART and referred to treatment in 15 intervention communities.

In summary, we found that of the 3,657 PLHIV who were referred for treatment, 3,282 (90%) linked to care, 3,066 (93%) of those initiated ART, and 2,951 (98%) of those who initiated ART were still on treatment at the end of the study out of those who were still alive. In addition, 2,854 (97%) of those still on ART had current VL results and 2,784 (98%) of those were virally suppressed at study end. Thus, of the 3,592 HIV-positive persons who were referred for treatment through BCPP and were alive at study end, 2,784 (78%) were documented to be on ART and had undetectable HIV-1 RNA (Fig 3).

## Discussion

Over the course of the BCPP study, we followed a longitudinal cohort of 3,657 newly identified or known HIV-positive persons who were not on treatment. Individual level tracking using a unique identifier allowed us to trace individuals through each step of the cascade to identify gaps and target interventions where needed to improve uptake and retention. Overall, 78% of referred persons who initiated ART and remained alive were retained and virally suppressed at study end.

Ninety percent of newly identified and known persons living with HIV not on ART linked to care, which is much higher than linkage rates from most community testing studies [4, 5]. A small proportion (11%) of those who linked were found on treatment at clinics outside their community of residence through EMR and laboratory systems. There are no known published studies that have tracked and verified referred persons obtaining care and treatment at non-referred clinics [5]. Those who never linked were more likely to be younger, males, newly identified HIV-positive, employed, and have more education. These findings and others [3, 32, 33] suggest that this group may need to be targeted for interventions at the time of referral and may need specific interventions targeted to their circumstances. For example, community and workplace models of ART service delivery may be more effective for working persons who cannot attend facilities during clinic hours or for persons with stigma or privacy concerns [34–36]. It is also possible that some of the employed individuals were receiving treatment from private doctors, but we were unable to obtain documentation of private care.

High rates of treatment initiation and retention on treatment were observed in this study. Interventions such as Rapid/Same-day ART initiation, 2–3 month dispensing of anti-retrovirals (ARVs) and 6-month clinic consultations likely contributed to these results. For example, Rapid/Same-day ART start reduced the number of pre-ART visits (from an average of 3–4 to 1) for adherence counseling and allowed initiation to occur before baseline lab measures were returned, which significantly reduced time from referral to initiation [37]. Six-month consultations and 2–3 month dispensing of ARVs also reduced the number of clinic visits, which was likely more convenient and less costly for patients. Rapid/Same-day start and other differentiated models of service delivery have been shown to increase the number of PLHIV initiating ART and to improve or maintain high rates of retention on ART [27, 35, 38, 39]. Other interventions which improved service delivery, including efforts to track and reschedule those due for VL testing and reduce turn-around-time of VL results from the lab may have contributed to the strong VL coverage and suppression rates. A small proportion (7%) of persons who linked did not initiate treatment; these individuals could not be identified as on ART through the MoHW EMRs or data systems.

Tracking and tracing efforts were implemented throughout the study to find those persons who never linked, linked but did not initiate treatment, or were late for appointments, drug pick-ups, VL tests or were LTFU. These efforts resulted in finding and returning to care or documenting outcomes of most of the persons who were late, missing or lost, leaving only 61 persons LTFU at the end of the study. We identified data quality issues that impacted retention rates, including incomplete records, un-entered data from registers/paper files, and data entry errors. The true number of patients lost in this study was much smaller once electronic tracing and data cleaning were completed, so the burden of people who required more resource-intensive tracing by phone or home visits was a small proportion of those initially appearing as LTFU. Tracking and tracing those LTFU in other studies showed similar findings; many persons are still active in care/on treatment but not properly documented and a smaller number are truly lost [23, 24]. Together, these studies suggest that apparent low retention rates may be an overestimate of the true number of persons out of care/off treatment. In addition, as countries adopt unique identifiers, it will be easier to locate and document persons attending other clinics. These changes will result in more accurate retention estimates and indicate which interventions are needed to return patients to treatment.

Many of the interventions implemented in this study are becoming standard of care in MoHW clinics serving PLHIV in sub-Saharan Africa; implementation and effectiveness should be monitored closely. The main challenges we observed when scaling up these interventions were obtaining accurate data from the clinics, as we encountered issues with poor data quality including poor documentation of patient data, lack of data entry into electronic data systems from registers and paper forms, and data entry errors. Resource limited settings often contend with issues such as power outages, broken equipment, and limited staff to keep up with the workload. We added an additional staff person to the study clinics to address these issues and ensure that all patient encounters were documented and entered correctly. However, additional efforts were required throughout the study to assist with data cleaning to ensure outcomes were correct and to find missing patients or data. As country programs scale-up these interventions, sufficient staff and resources for data management, monitoring and evaluation should be considered as necessary components of the interventions.

There were several limitations to this study including the lack of a comparison group. This study design had rolling enrollment over time, which prohibited us from following all persons in the cohort for the same amount of time. In addition, the study was conducted in peri-urban and rural communities, so it is not clear how generalizable these findings are to urban areas.

## Conclusions

This study achieved high rates of linkage, treatment initiation, retention and VL suppression in a cohort of newly identified and known PLHIV not on ART referred for treatment from community testing. Individually linked data allowed us to see where losses were occurring along the clinical cascade and where and to whom to target interventions over the course of the study. A small percent of PLHIV needed support with linkage and retention and tracking and tracing interventions were effective for identifying those who needed more resource intensive follow-up. These results were achieved in MoHW public clinics with support for strengthening service delivery models; results can be generalized to settings with high HIV prevalence and high levels of testing and treatment coverage. Ensuring these gaps are addressed with feasible and effective interventions is critical for retaining PLHIV on treatment and reducing new infections.

## Supporting information

S1 File (PDF)

S2 File (ZIP)
