# Supplementary material for: Finding, treating and retaining persons with HIV in a high HIV prevalence and high treatment coverage country: Results from the Botswana Combination Prevention Project
Source: PLoS One. 2021 Apr 21;16(4):e0250211. doi: 10.1371/journal.pone.0250211 (PMC8059857; doi:10.1371/journal.pone.0250211)
Supplement: S1 File — (PDF) [file pone.0250211.s001.pdf]

TEBELOPELE VOLUNTARY COUNSELING AND TESTING CENTRE

HIV COUNSELLING AND TESTING RECORD (BOTSWANA Combination Prevention Project, 23 August 2016)

INDIVIDUAL INTAKE FORM	
**Instructions:**<br>*After completing the enumeration form, complete this client intake form for all individuals in the household or at the mobile testing location who are ≥16 years of age and who give permission for you to talk to them about HIV testing and counseling. Words in italics or light font are instructions for interviewer and should not be read out loud. Words in boldface type are questions for the client.*	
**Date of interview (DD/MM/YYYY)**	
**Counselor code**	
**We would now like to talk to you about HIV testing and counseling and ask some questions about you and your background. Do you give permission for us to proceed?**	□ Yes *(go to section A, question 1)*<br>□ No *(go to refusal question R1)*
**R1. Refusal ID number (*Obtain this number from the list of IDs for participants refusing the intake form. Do not use client unique ID number.*)**	_ - _ _ - _ _ _ _
**R2. Testing and counseling site**	□ In home *(go to R2a)*<br>□ Mobile unit *(go to R3)*<br>□ Tent *(go to R3)*
**R2a. Specify enumeration form household member ID**	_ _ _ _ _ _ - _ _ - _ ***(specify HH number and member letter)***
**R3. Gender**	□ male<br>□ female
**R4. What is your age?**	_ _ _
**R5. We respect your decision to decline. It would help us improve the Ya Tsie study if you could tell me the main reason why you do not want to participate.**	□ I don't have time due to household duties<br>□ I don't have time due to work commitments<br>□ I don't have time for other reasons, specify other reasons ____________<br>□ I don't want to answer the questions<br>□ My family member(s) don't want me to answer the questions<br>□ I am afraid my information will not be private<br>□ I already know I am HIV+<br>□ I already know I am HIV-<br>□ I already know my HIV status (participant did not disclose status to interviewer)<br>□ Other, specify: ___________________________<br>□ Declined to answer

A. Household location information	
1. **BCPP community name**	
2. **Plot unique ID**	
**2a. Household unique ID**	
3. **Description of physical address** *Include ward (if known), location relative to roads and landmarks, and distinguishing information such as number and type of outbuildings, the color of the door, etc.*	________________<br>________________<br>________________
4. **GPS coordinates (record at main gate to household)**	
5. **CSO Number** *Record four nines (9999) if CSO number is not present at household.*	
**B. Client information**	
6. **Client unique ID**	
7. **First name**	
8. **Middle name**	
9. **Surname**	
10. **Is ________ (***state BCPP community currently in***) your community of residence?** *(community of residence is where individual spends, on average, ≥14 nights per month)*	□ Yes *(go to 10a-1)*<br>□ No *(go to 10a)*
**10a. Are you a part-time resident of _____ *(state BCPP community currently in*)? *(A person is a part-time resident if they spend between 3 and 13 nights per month in a community.)***	□ Yes *(go to 10a-1)*<br>□ No *(go to 10b)*
**10a-1. How long have you lived in this community?**	□ less than 6 months (go to 11)<br>□ 6 months to 12 months (go to 11)<br>□ 1 to 5 years (go to 11)<br>□ more than 5 years (go to 11)

**10b. What is your community of residence?**	
11. **Are you a Botswana citizen?**	□ Yes *(go to 11a)*<br>□ No *(go to 11c)*
**11a. Do you have an Omang number?**	□ Yes *(go to 11b)*<br>□ No *(go to 11d)*
**11b. Omang number**<br>*The Omang number should be transcribed from the Omang card.*	______________________________ *(go to 12)*
**11c. If not a citizen, are you legally married to a Botswana citizen?**<br>*A marriage certificate must be shown for a "yes" answer.*	□ Yes *(go to 11d)*<br>□ No *(go to 11d)*
**11d. Do you have a passport?**	□ Yes *(go to 11e)*<br>□ No *(go to 12)*
**11e. Passport number**	______________________________ *(go to 12)*
12. **Date of Birth (DD/MM/YYYY)**	
13. **Age**	
14. **Sex**	□ Female *(go to 14b)*<br>□ Male *(go to 15)*
14b. For female participants only: **Are you pregnant?**	□ Yes *(go to 15)*<br>□ No *(go to 15)*<br>□ Don't know *(go to 15)*
15. **Would you be willing to provide a phone number we can call in case we need to contact you for any follow up?**	□ Yes *(go to 15a)*<br>□ No *(go to 16)*<br>□ Don't have a phone *(go to 16)*
**15a. Please give me the best phone numbers to reach you at.**	1. Telephone Number: _______________<br>*2.* Telephone Number:________________<br>*(go to 15b)*

**15b. If we cannot reach you at this number, is there a family/friend we can call?**	□ Yes *(go to 15c)*<br>□ No *(go to 16)*
**15c. Contact information of family/friend who will know where you are if we can't reach you (must be 18 years of age or older)?**	1. Name:________________________________<br>2. Relation:______________________________<br>3. Telephone Number:_____________________<br>**4.** Telephone Number 2:____________________<br>**5.** Address: ______________________________
16. **Testing and counseling site**	□ in home *(go to 16a)*<br>□ mobile unit *(go to 17)*<br>□ tent *(go to 17)*
**16a. Specify household visit number**	□ Visit 1 ***(go to 16b)***<br>□ Visit 2 ***(go to 16b)***<br>□ Visit 3 ***(go to 16b)***
**16b. Specify enumeration form household member ID**	_ _ _ _ _ _ **-** _ _ **-** _ ***(specify HH number and member letter)***
**C. Demographics and Risk Factors**	
17. **What level of education have you completed? *(Tick only one answer choice)***	□ None<br>□ Non formal<br>□ Primary<br>□ Secondary<br>□ Tertiary (Higher than secondary, such as vocational college or university)
18. **What is your current employment status? *(Tick only one answer choice)***	□ Full-time employed<br>□ Part-time employed<br>□ Seasonal or intermittent employment<br>□ Informal self-employment<br>□ Student<br>□ Retired<br>□ Not working (non-student, not retired)<br>□ Don't want to answer
19. **What is your current marital status?**	□ Single/Never married<br>□ Cohabitating<br>□ Married<br>□ Divorced or formally separated<br>□ Widowed<br>□ Don't want to answer
20. **Now I would like to ask you about how frequently you drink alcohol. In the past THREE months, how often have you had a drink containing alcohol?**	□Never<br>□Monthly or less<br>□2-4 times per month<br>□2-3 times per week<br>□4 or more times per week

Now I will ask some questions about sex and sex partners. Some of these questions may make you uncomfortable; however, please remember that your answers are confidential and it is really important for us to get the most honest answer you can give us. In this set of questions, when I say sex, I mean vaginal or anal sex. I do not mean oral sex, kissing, or touching with hands. When I say a partner, I mean anyone you might have had sex with. Partners can be your husband, wife or wives, girlfriends, boyfriends, friends, casual partners, prostitutes, or someone you may have met at a bar, or at a wedding or other special events, etc.	
21. **Have you ever had sex?**	□ Yes *(go to 22)*<br>□ No *(go to 27)*
22. **Have you had sex in the past 12 months?**	□ Yes *(go to 23)*<br>□ No *(go to 27)*
23. **How many sex partners have you had in the past 12 months?** *(enter number as 01, 02, etc.)*	_ _ *if 1, complete 24a-d;*<br>*if 2, complete 24a-d and 25a-d*<br>*if 3 or more, complete 24a-d, 25a-d, and 26a-d*
**Now I am going to ask you some questions about sexual partners you have had in the <u>last 12 months</u>.**	
**24a. I would like to start by asking some questions about your most recent sexual partner. What is your relationship with this partner?**	□ Spouse (husband/wife)<br>□ Cohabitating partner<br>□ Boyfriend/Girlfriend<br>□ Casual (known) sex partner<br>□ One time partner (previously unknown)<br>□ Commercial sex worker<br>□ Other, specify: ___________________<br>□ Decline to answer
**24b. What is this partner's HIV status?**	□ HIV Positive *(skip to 24d)*<br>□ HIV Negative *(go to 24c)*<br>□ I am not sure *(go to 24c)*<br>□ Decline to answer *(go to 24c)*
**24c. Has this partner been tested for HIV in the last 12 months?**	□ Yes (*go to 24d*)<br>□ No *(go to 24d)*<br>□ Don't know *(go to 24d)*
**24d. Does this partner live in this community?**<br>*The partner lives in the community if he/she usually spends 14 nights or more in the community each month.*	□ Yes<br>□ No<br>□ Don't know
**25a. Now I am going to ask you about your second most recent sexual partner. What is your relationship with this partner?**	□ Spouse (husband/wife)<br>□ Cohabitating partner<br>□ Boyfriend/Girlfriend<br>□ Casual (known) sex partner<br>□ One time partner (previously unknown)<br>□ Commercial sex worker<br>□ Other, specify: ___________________<br>□ Decline to answer

**25b. What is this partner's HIV status?**	□ HIV Positive *(skip to 25d)*<br>□ HIV Negative *(go to 25c)*<br>□ I am not sure *(go to 25c)*<br>□ Decline to answer *(go to 25c)*
**25c. Has this partner been tested for HIV in the last 12 months?**	□ Yes (*go to 25d*)<br>□ No *(go to 25d)*<br>□ Don't know *(go to 25d)*
**25d. Does this partner live in this community?**<br>*The partner lives in the community if he/she usually spends 14 nights or more in the community each month.*	□ Yes<br>□ No<br>□ Don't know
**26a. Now I am going to ask you about your third most recent sexual partner. What is your relationship with this partner?**	□ Spouse (husband/wife)<br>□ Cohabitating partner<br>□ Boyfriend/Girlfriend<br>□ Casual (known) sex partner<br>□ One time partner (previously unknown)<br>□ Commercial sex worker<br>□ Other, specify: ___________________<br>□ Decline to answer
**26b. What is this partner's HIV status?**	□ HIV Positive *(skip to 26d)*<br>□ HIV Negative *(go to 26c)*<br>□ I am not sure *(go to 26c)*<br>□ Decline to answer *(go to 26c)*
**26c. Has this partner been tested for HIV in the last 12 months?**	□ Yes (*go to 26d*)<br>□ No *(go to 26d)*<br>□ Don't know *(go to 26d)*
**26d. Does this partner live in this community?**<br>*The partner lives in the community if he/she usually spends 14 nights or more in the community each month.*	□ Yes<br>□ No<br>□ Don't know
**D. HIV testing history**	
**Many people have had a test to see if they have HIV. Now I am going to ask you some questions about whether you have been tested for HIV and, if you have been tested, whether you received the results. Please remember that all of your answers are confidential.**	
**27. Have you ever previously been tested for HIV?**	□ Yes *(go to 28)*<br>□ No *(go to Part F – HIV testing and counseling)*

**28. Where did you last undergo HIV testing?**	□ TVCT in this community<br>□ TVCT outside of this community<br>□ Antenatal care at Public Healthcare Facility<br>□ Other care (not antenatal) at Public Healthcare Facility<br>□ Antenatal care at Private Healthcare Facility<br>□ Other care (not antenatal) at Private Healthcare Facility<br>□ Door to door projects<br>□ Other VCT site<br>□ Other<br>□ Don't remember
**29. Is a record of your last HIV test available to review today? *(for HIV+ persons, other documentation of HIV status, such as a health card or pill bottle, is acceptable)***	□ Yes *(go to 29a)*<br>□ No *(go to 29c)*<br>□ Decline to answer *(go to 29c)*
**29a. Recorded date of previous test: *(Only record if written documentation of test date. Do not record self-reported test dates.)***	___/____/____ (day/month/year) *(go to 29b)*
**29b. Recorded result of previous test:**	□ HIV+ (*go to 29d*)<br>□ HIV- (*go to 29e*)<br>□ Indeterminate (*go to Section E*)
**29c. What was the result of your last HIV test?**	□ HIV+ (*go to 29d*)<br>□ HIV- (*go to 29e*)<br>□ Indeterminate (*go to Section E*)<br>□ Decline to answer (*go to Section E*)
**29d. Have you ever attended a health clinic for HIV care?**	□ Yes (*go to 29d-1*)<br>□ No (*go to Section E*)<br>□ Decline to answer *(go to Section E)*
**29d-1. What is the name of the clinic you most recently visited for HIV care?**	____________________________ (*go to 29d-2*)
**29d-2. Do you have a health card available to review today?** *Check yes if client shows card to counselor, check no if client does not show card.*	□ Yes (*go to 29d-2a*)<br>□ No (*go to 29d-4-0*)<br>□ Decline to answer (*go to 29d-4-0*)
**29d-2a. Is there documentation that the client attended the clinic reported in 29d-1?**	□ Yes (*go to 29d-3*)<br>□ No (*go to 29d-3*)<br>□ Decline to answer (*go to 29d-3*)

**29d-3. What is the next scheduled appointment date?** *Record most recent scheduled appointment date (even if date has passed)*	___/____/____ (day/month/year) *(go to 29d-4-0)*
**29d-4-0. Have you ever taken ART?**	□ Yes (*go to 29d-4*)<br>□ No (*go to Section E*)<br>□ Decline to answer (*go to Section E*)
**29d-4. Are you currently taking ART?**	□ Yes (*go to 29d-4a*)<br>□ No (*go to Section E*)<br>□ Decline to answer (*go to Section E*)
**29d-4a. Is there documentation that the client is on ART? *(A pill bottle or ART refill card is acceptable documentation of ART)***	□ Yes (*go to Section E*)<br>□ No (*go to Section E*)<br>□ Decline to answer (*go to Section E*)
**29e. When was the HIV- test result obtained? *If fewer than 3 months have elapsed since test date, test is within 3 months.***	□ Less than 3 months ago (*go to Section E)*<br>□ More than 3 months ago (*go to Section E*)<br>□ Test date not recorded (*go to Section E)*

E. HIV testing and referral instructions

Use the participant responses in section D and the job aid "HIV and CD4: Who to Test" to determine whether testing is recommended for the participant.

HIV testing is recommended for all clients who:

- ***Do not have documentation of HIV status (including self-reported HIV+)***
- ***Tested HIV- more than three months ago***
- ***Are pregnant and are HIV- or HIV unknown***
- ***Request an HIV test***

For clients who request a HIV test or for whom an HIV test is recommended, go to question 30-1.

For known HIV+ clients who are not on ART, go to question 30-2.

For clients who tested HIV- within the last three months and are not pregnant and have not requested a test, go to question 33.

F. HIV testing and counseling

Complete this section for ALL CLIENTS, following skip patterns carefully

**30-1. Do you consent to HIV testing and counseling today? (Skip CD4 Q)** If you test positive we will do a CD4 test and give you the result to share with medical providers.	□ Yes *(perform testing and record testing details in 30b)*<br>□ No *(go to question 30a)*
**30-2. (Skip) Because you have previously tested HIV+ and have not yet started treatment, we recommend a CD4 test to measure how strong your body is. If you choose to have a CD4 test performed today, we will give you your CD4 test result to take to the clinic. At the clinic, you might be eligible for treatment through the MOH or through the Ya Tsie study. Do you consent to a CD4 test today?**	□ Yes *(go to 31)*<br>□ No *(go to question 31b)*
**30a. What is the main reason you did not want HIV testing as part of today's visit?** *(Tick best answer choice. After participant answers, thank them for their time. Save form and close.)*	□ I already know I am HIV positive (*Go to question 30-2)*<br>□ I recently tested (know status is negative) (*Go to question 33)*<br>□ I don't believe I am at risk of getting HIV (*Go to question 33)*<br>□ I am afraid to find out the result (*Go to question 33)*<br>□ I am afraid of what others would think of me (*Go to question 33)*<br>□ Family/friends did not want me to get an HIV test (*Go to question 33)*<br>□ I didn't have time due to work (*Go to question 33)*<br>□ I didn't have time due to family obligations (*Go to question 33)*<br>□ My sexual partner didn't want me to get an HIV test (*Go to question 33)*<br>□ I am not sure (*Go to question 33)*<br>□ Decline to answer (*Go to question 33)*
**30b. Was a Determine test performed?**	□ Yes *(go to 30b-1)*<br>□ No *(go to 30i)*
**30b-1. Enter the lot number for the first or only Determine test.**	___________________ *(go to 30b-2)*

**30b-2. Enter the expiry date for the first or only Determine test.** *(DD/MM/YYYY)*	_ _/_ _/_ _ _ _ *(go to 30b-3)*
**30b-3. Enter the result for the first or only Determine test.**	□ R *(go to 30c)*<br>□ NR *(go to 30c)*<br>□ INV *(go to 30c)*
**30c. Was a UNI-GOLD test performed?**	□ Yes *(go to 30c-1)*<br>□ No *(go to 30i)*
**30c-1. Enter the lot number for the first or only UNI-GOLD test.**	___________________ *(go to 30c-2)*
**30c-2. Enter the expiry date for the first or only UNI-GOLD test.** *(DD/MM/YYYY)*	_ _/_ _/_ _ _ _ *(go to 30c-3)*
**30c-3. Enter the result for the first or only UNI-GOLD test.**	□ R *(go to 30d)*<br>□ NR *(go to 30d)*<br>□ INV *(go to 30d)*
**30d. Was a <u>second</u> Determine test performed?**	□ Yes *(go to 30d-1)*<br>□ No *(go to 30e)*
**30d-1. Enter the Determine lot number for the <u>second</u> Determine test**	___________________ *(go to 30d-2)*
**30d-2. Enter the expiry date for the <u>second</u> Determine test** *(DD/MM/YYYY)*	_ _/_ _/_ _ _ _ *(go to 30d-3)*
**30d-3. Enter the <u>second</u> Determine result**	□ R *(go to 30e)*<br>□ NR *(go to 30e)*<br>□ INV *(go to 30e)*
**30e. What is the total number of Determine test strips used?**	___________________ *(go to 30f)*
**30f. Was a <u>second</u> UNI-GOLD test performed?**	□ Yes *(go to 30f-1)*<br>□ No *(go to 30g)*
**30f-1. Enter the UNI-GOLD lot number for the <u>second</u> UNI-GOLD test.**	___________________ *(go to 30f-2)*
**30f-2. Enter the expiry date for the <u>second</u> UNI-GOLD test** *(DD/MM/YYYY)*	_ _/_ _/_ _ _ _ *(go to 30f-3)*
**30f-3. Enter the <u>second</u> UNI-GOLD result**	□ R *(go to 30g)*<br>□ NR *(go to 30g)*<br>□ INV *(go to 30g)*
**30g. What is the total number of UNI-GOLD test strips used?**	___________________ *(go to 30h)*

**30h. Was the sample sent for re-testing?**	□ Yes *(go to 30i)*<br>□ No *(go to 30i)*
**30i. Today's results:**	□ HIV+ *(go to 31)*<br>□ HIV- *(go to 31-e)*<br>□ Indeterminate *(refer to clinic for follow-up testing; go to 31d)*<br>□ Not tested *(e.g. insufficient sample; go to 31)*
**31. (Answer no and Skip to 31c) For participants who tested positive or are known HIV+ and not on ART, was the sample of sufficient volume and quality to run a CD4 test today?**	□ Yes *(go to 31a)*<br>□ No *(go to 31c)*
**31a. Was CD4 test performed?**	□ Yes *(go to 31a-1)*<br>□ No *(go to 31b)*
**31a-1. Enter the PIMA cartridge lot number.**	___________________ *(go to 31a-2)*
**31a-2. Enter the PIMA cartridge expiration date.**	_ _/_ _/_ _ _ _ *(go to 31a-3)*
**31a-3. Was sample sent for EQA?**	□ Yes *(go to 31a-4)*<br>□ No *(go to 31a-4)*
**31a-4. CD4 test result**	_ _ _ _ *(go to 31c)*
If a CD4 test was not performed because the client did not wish to continue with testing, read question 31b. If testing was not performed because of problems with PIMA machine function, then do not read question 31b. Mark appropriate answer choice.<br><br>**31b. What is the main reason you did not wish to have a CD4 test performed as part of today's visit?**	□ Privacy/disclosure concerns *(go to 31c)*<br>□ Do not believe it's important *(go to 31c)*<br>□ Do not have time *(go to 31c)*<br>□ Other, specify:_____________________ *(go to 31c)*<br>□ Don't want to answer *(go to 31c)*<br>□ Question not applicable: test not performed because PIMA machine malfunctioned *(go to 31c)*
**31c. Name of clinic referred to**	*(go to 31d)*
**31d. Appointment date** *(DD/MM/YYYY)*	_ _/_ _/_ _ _ _ *(go to 31e)*

**31e. Describe sample collection experience. *Check all that apply. Then go to 32***	□ Sample volume sufficient (>250 ul)<br>□ Sample did not have visible clots<br>□ Sample volume insufficient (<250 ul)<br>□ Sample clotted<br>□ Extended collection time<br>□ Could not draw blood (e.g. fingers callused, could not break skin with lancet; fingers too cold)<br>□ Other, specify: ___________________________
**32. Did testing and counseling occur through couples testing today?**	□ Yes *(go to 32a)*<br>□ No *(go to 33)*
**32a. What is the unique identification number for the other member of the couple?**	
**33. Does the client currently have any of the following symptoms?** *(check all that apply then go to 34)*	□ cough > 2 weeks<br>□ fever > 2 weeks<br>□ enlarged lymph nodes *(swelling in the neck, armpit, or groin)*<br>□ coughing up blood<br>□ night sweats, with enough sweating to soak the sheets<br>□ unexplained weight loss<br>□ none of the above symptoms reported
**34. Have any of the client's family members been diagnosed with tuberculosis?**	□ Yes *(go to 35)*<br>□ No *(go to 35)*<br>□ Don't know *(go to 35)*
**35. Client referred FOR:** *(check all that apply then go to 36)*	□ Circumcision<br>□ Cervical screening<br>□ STI Screening<br>□ Family planning<br>□ TB Screening<br>□ Couple testing<br>□ Retesting (participants with indeterminate result)<br>□ PMTCT/ANC<br>□ HIV Care and Treatment<br>□ Supportive Counseling<br>□ Psycho-social support / Social welfare services
**36. Client referred TO:** *(check all that apply then go to Section G)*	□ Public/Private Health Facility<br>□ Religious Institution<br>□ PLWH/A Association<br>□ Social Welfare facilities<br>□ Youth Friendly Services
**G. Follow-up**	

For men who are HIV negative or unknown (declined testing) ask questions 38 - 38a.<br><br>***For persons with an indeterminate test result, read items 40 and 40a.***
For all participants: Thank you for your participation today. We appreciate the time you took to answer these questions and we will follow up with you if needed.
Men who are HIV negative or unknown status (ask questions 38 -38a).

**38. Male circumcision is the removal of the foreskin of the penis. Here is a diagram showing the difference between a circumcised and an uncircumcised penis. We are offering free transportation to safe male circumcision services as part of Ya Tsie.**<br>Male circumcision helps protect men from getting HIV and some other infections which are spread by sex, and also helps protect their partners.<br><br>**Unless you decline, a Ya Tsie male circumcision staff member may contact you by phone or home visits to provide information about circumcision and answer any questions. If we call and miss you, the caller will not leave any detailed message, only their contact information. If someone asks, the caller will say we are doing mobilization for men's health.**	□ Participant did not decline further contact *(Tablet should display man's physical address and phone number; counsellor should write these on the SMC demand creation register. Then STOP interview. Thank the client for his time. Give counseling and referrals as appropriate.)*<br><br>□ Participant declined further contact *(Go to 38a.)*

**38a. Okay. Could you tell me the main reason why at this time you are not interested in learning more?**<br><br>*(Tick one answer. Then, unless the answer is "already circumcised", read the following text.)*<br><br>*"Thank you for sharing your concerns with me. Understanding that you do not want to discuss circumcision now, if you want to later, any HTC staff person can help you connect with an SMC representative at a later time."*	□ Already circumcised<br>□ Afraid of pain<br>□ Do not believe it protects men against HIV<br>□ Not acceptable to the other men in my community<br>□ Not acceptable to women in my community<br>□ Believe I am too old<br>□ Not ready to make a decision about circumcision<br>□ Plan to go for circumcision, but at a later time<br>□ No specific plan for circumcision, but too busy to go at this time<br>□ Not too old, but do not believe I am at risk for HIV for a different reason.<br>□ Other (specify:_______________)<br><br>***STOP interview. Thank the client for his time.***
For persons who have an indeterminate test result, read items 40 and 40a.	
**40. Today your HIV test result was indeterminate. This means that it is not possible to determine from today's test whether you are infected with HIV. It is important that you return to the clinic in two weeks for another test. We may contact you by phone calls and home visits to remind you to return to the clinic for another test.**	
**40a. If your follow-up test shows that you are HIV infected, counselors are available to help you begin HIV care and treatment at the local health clinic.**	
