# Supplementary material for: Finding, treating and retaining persons with HIV in a high HIV prevalence and high treatment coverage country: Results from the Botswana Combination Prevention Project
Source: PLoS One. 2021 Apr 21;16(4):e0250211. doi: 10.1371/journal.pone.0250211 (PMC8059857; doi:10.1371/journal.pone.0250211)
Supplement: S2 File — (ZIP) [file pone.0250211.s002.zip › ClinicalCascade DataReleasePackage/BCPP_clinicalcascade_SASPgmExample.docx]

*************************************************************************************

* This SAS program may be used to reproduce tables 1-2 and figures 1-2 in the manuscript titled: *

* Finding, treating and retaining persons with HIV in a high HIV prevalence and high treatment *

* coverage country: Results from the Botswana Combination Prevention Project *

*************************************************************************************

dm 'clear output; clear log'; options pageno=**1**;

*import csv data file named “bcpp_clinicalcascade” and create a temp sas dataset named, cascade;

/************************************************************/

*Linkage to care of persons referred to treatment;

/************************************************************/

*Analysis: Table 1, demographics;

**proc** **freq** data = cascade;

table (agecategory intakehivstat education employment maritalstatus)*gender / norow nopercent;

**run**;

*Analysis: Table 2, linkage to care – sample covariates to demonstrate in code;

*Sex;

**proc** **freq** data=cascade;

tables gender*linked/ nocol nopercent;

**run**;

**proc** **glimmix** data=cascade;

nloptions tech=nrridg;

class community gender;

model linked=gender /dist=bin link=logit solution;

lsmeans gender /cl ilink;

estimate 'female vs. male' gender **1** -**1** /cl exp;

random community;

**run**;

*Age;

**proc** **freq** data=cascade;

tables agegrp*linked/ nocol nopercent;

**run**;

**proc** **glimmix** data=cascade;

nloptions tech=nrridg;

class agegrp community;

model linked=agegrp /dist=bin link=logit solution ;

estimate '25-34 vs. 16-24' agegrp -**1** **1** **0** /cl or;

estimate '35-64 vs. 16-24' agegrp -**1** **0** **1** /cl or;

lsmeans agegrp /cl ilink;

random community;

**run**;

*Analysis: linked to the clinic of community residence;

**proc** **freq** data=cascade;

tables linkedtocpc / nocol nopercent;

where linked = **1**;

**run**;

*Analysis: kept appointment versus linked later to clinic of community residence;

**proc** **freq** data=cascade;

tables linked7d / nocol;

where linked = **1** and linkedtocpc = "1";

**run**;

/************************************************************/

*Section: ART initiation and retention among persons linking to care;

/************************************************************/

*Analysis: Initiated treatment among those who linked to care;

**proc** **freq** data = cascade;

tables artstart;

where linked = **1**;

**run**;

*Analysis: Deaths among those who linked but did not initiate treatment;

**proc** **freq** data = cascade;

tables isdead;

where linked = **1** and artstart = **0**;

**run**;

*Analysis: Retained on ART at study end;

**proc** **freq** data = cascade;

tables retainedonart;

where artstart = **1** and isdead = "0";

**run**;

/************************************************************/

*Section: Viral load testing and viral suppression;

/************************************************************/

*Analysis: Current viral load test at study end;

**proc** **freq** data = cascade;

tables vltest;

where retainedonart = "Yes";

**run**;

*Analysis: Viral suppression at study end;

**proc** **freq** data = cascade;

tables vlsuppress;

where retainedonart = "Yes";

**run**;
